# Takayasu arteritis in peripartum period—A clinical paradox

**DOI:** 10.1002/ccr3.7128

**Published:** 2023-03-29

**Authors:** Lokesh Koumar Sivanandam, Naresh Kumar Reddy, Ratnasree Vaddepalli, Vivek Sanker, Anjuman Amina Mansoor, Umang Gupta

**Affiliations:** ^1^ Sri Lakshmi Narayana Institute of Medical Sciences Puducherry India; ^2^ Team Erevnites Puducherry India; ^3^ Internal Medicine, Sri Lakshmi Narayana Institute of Medical Sciences Puducherry India; ^4^ Angeles University Foundation Pampanga Philippines; ^5^ Noorul Islam Institute of Medical Sciences Trivandrum India; ^6^ MBBS, JIPMER Puducherry India; ^7^ Nepalgunj medical college Nepalgunj Banke Nepal

**Keywords:** case report, peripartum, pregnancy, Takayasu arteritis

## Abstract

Takayasu arteritis is a primary systemic vasculitides occurring among women in the childbearing age group. This interaction between TA and pregnancy is an area of interest that has to be addressed. Preconception and antepartum management of arterial hypertension and TAK disease activity is important to improve maternal and fetal outcomes.

## Graphical abstract

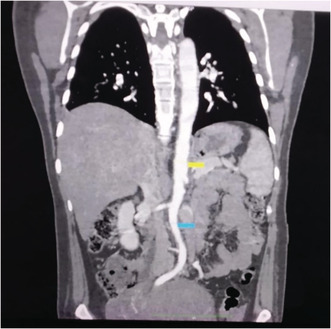

## 1 INTRODUCTION

Takayasu arteritis is a granulomatous inflammation of the aorta and its major branches. It can present as an isolated, atypical, and/or catastrophic disease. The disease has been reported worldwide, although it appears to be more prevalent in Asians. We report the case of a 29‐year‐old pregnant lady presented with TA.

Takayasu arteritis (TA), first reported by Japanese ophthalmologist Mikito Takayasu, is a chronic inflammatory disease of medium and large arteries of unknown etiology. A higher prevalence of this disease is seen in Asian countries such as Japan and India and South America, the regional predilection also reflects in the presentation of the disease.^1^ It has female preponderance and manifests mostly in the second to third decade. Patients initially present with constitutional symptoms but later progress to develop symptoms related to vascular damage.

A majority of the population affected by the disease are women of childbearing age, and the interplay between vasculitis such as TA, and pregnancy becomes an area of attention. Studies have reported poor obstetric outcomes in patients with TA, with the most common complication being hypertension.^2^ On the other hand, due to TA's tardy diagnosis women conceive without being aware of their condition.^3^ Here, we describe a case of a 29‐year‐old pregnant woman who presented for safe confinement at the 40th week of gestation with an uneventful antepartum period, when TA was incidentally diagnosed.

## 2 CASE REPORT

A 29y old female G4P3L2A0 of 40 weeks of gestational age came for safe confinement in our hospital and was admitted to Obstetrics and gynecology department. She underwent emergency lower segment caesarean section (LSCS) because of severe oligohydramnios (Amniotic Fluid Index<3 cm), the baby and mother were in good health. She was taken over to the Department of Internal Medicine for further evaluation in view of the absent bilateral radial pulse, Blood pressure (BP) not recordable in both upper limbs. The patient has no significant past medical history and family history corresponding to TA. On examination, she is well built, with no pallor, icterus, clubbing, cyanosis, or pedal edema. On physical examination bilateral upper limb radial, brachial pulse not palpable, and BP not recordable. Bilateral carotid bruit present. Lower limb left dorsalis pedis pulse was not palpable and BP recordings were (right‐110/80 and left‐120/80). This case report has been reported in line with SCARE criteria.

**FIGURE 1:**
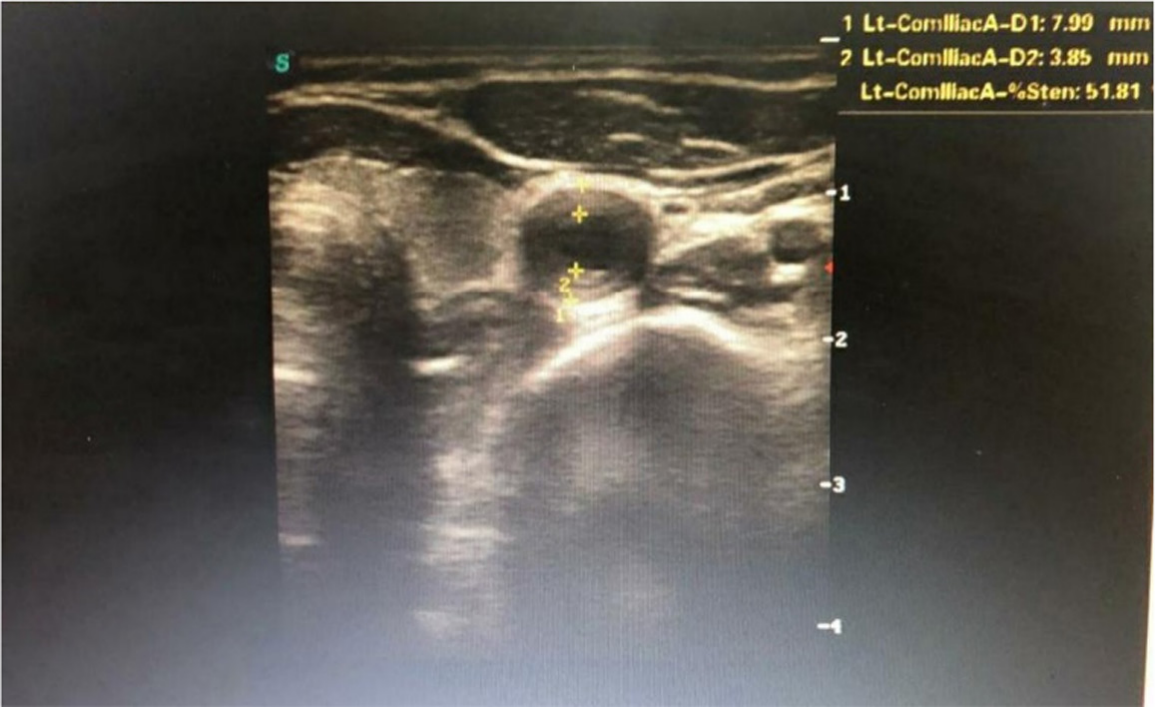
Macaroni sign: Seen on USG—smooth, homogenous, and circumferential thickening of arterial wall.

**FIGURE 2:**
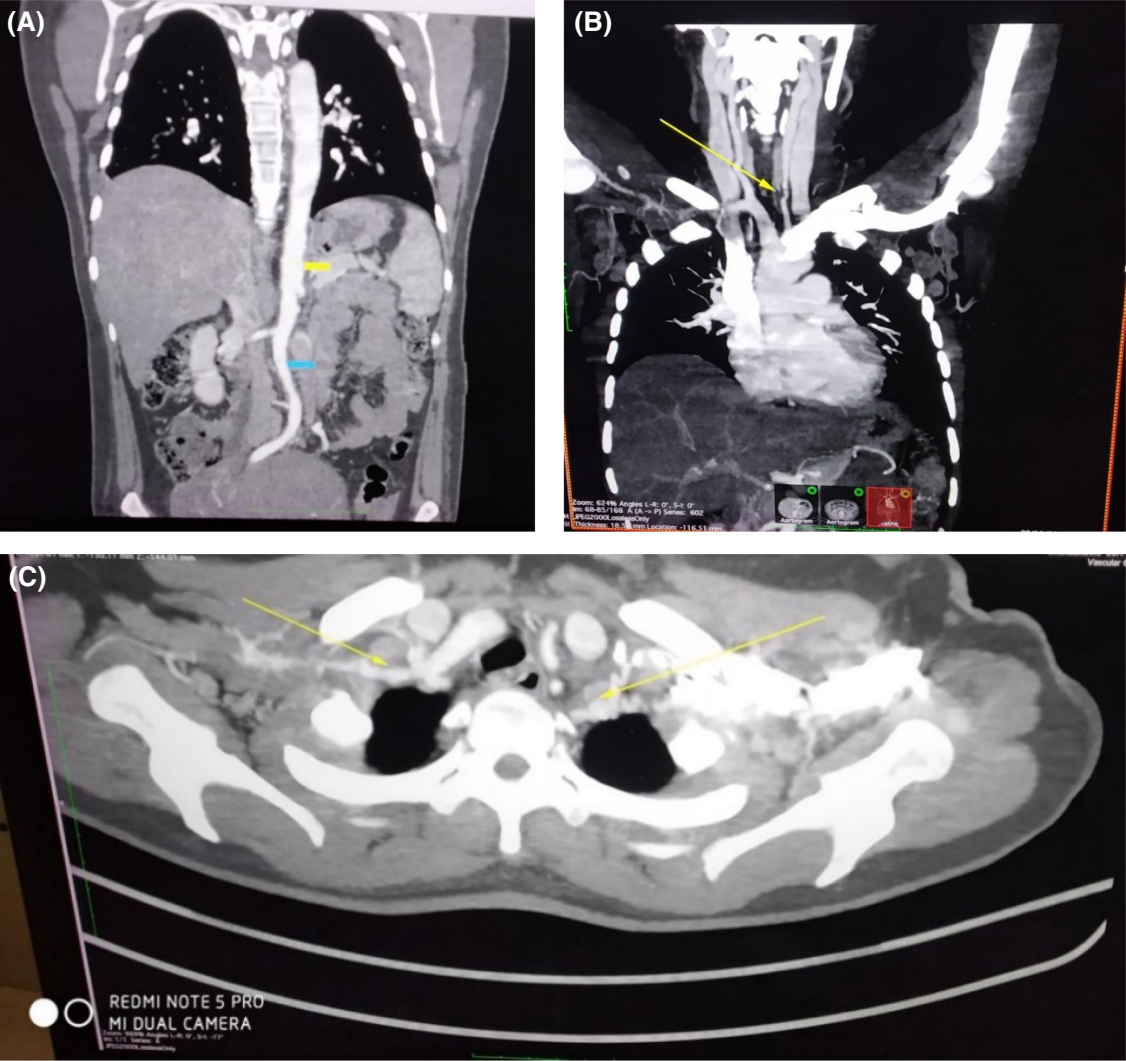
(A) Stenosis of the origin of the common carotid artery of the left (extension into external carotid artery). (B) Bilateral subclavian artery narrowing. (C) Carotid artery circumferential narrowing and left common iliac artery narrowing.

Her Ultrasonography (USG) carotid doppler showed left common carotid artery diffuse circumferential narrowing (60%) and lower limb doppler showed a “macaroni sign” (Figure 1). CT (Computed Tomography) aortogram showed right distal two‐thirds and left diffuse common carotid artery (Figure 2A), bilateral subclavian (Figure 2B), and infrarenal abdominal aorta, superior mesenteric, left common iliac artery circumferential narrowing without an aneurysm (Figure 2C). Electrocardiogram (ECG) was normal, and 2D‐Echo was normal with adequate Left Ventricle function. Laboratory findings showed elevated erythrocyte sedimentation rate (erythrocyte sedimentation rate 130 mm/hour) and C‐reactive protein level was 20 mg/dL at the time of admission. Considering these findings, the diagnosis was made as Takayasu Arteritis (type V).

The patient was started on high‐dose steroids (Tab Prednisolone 50 mg/day) with calcium and vitamin D3 cover and was discharged 5 days after delivery. In addition, in subsequent visits, ESR and CRP were done along with a screening ECHO (2D) and treatment was modified by tapering the steroids in view of declining inflammation. Tab Aspirin 75 mg was added as a secondary prophylaxis to prevent ischemic events. The patient was then followed up after 6 months; the ESR (20 mm/hr) and CRP were within normal limits with no new symptoms, and the ITAS (Indian Takayasu Arteritis Severity score) was low. With the above findings, the steroids were tapered and steroid‐sparing treatment (Azathioprine 50 mg BD) was initiated. The ITAS score and clinical improvement usually determine the duration of TA.

## 3 DISCUSSION

Takayasu arteritis is a chronic inflammatory disease of medium and large arteries, also known as “pulseless disease.” The aorta and its branches, pulmonary, coronary, and renal arteries are primarily affected. It is known to have a female preponderance and tends to manifest most commonly in the second to third decades of life.^1^ It occurs worldwide with the highest prevalence in Japan. In India, it is one of the common causes of renovascular hypertension. In Indian female patients with TA, the aortic arch and its branches are more commonly involved.

The disease has been attributed to various causes such as genetics, infections (namely, Mycobacterium tuberculosis), and autoimmune, although the exact etiopathogenesis is still unknown.^1^ The result of the process is vessel wall inflammation and chronic fibrosis with the formation of inflammatory aneurysms^4^ and eventually, stenosis and thrombosis of affected arteries,^5^ which gives rise to symptoms of ischemia.

The symptomatology of the disease varies by geographical region.^6^ For example, India has higher rates of hypertension, abdominal aorta involvement, and higher occurrence among the male population as compared to Japan.^6^ The patient may be asymptomatic to start with, as seen in the above case, which often leads to a delay in diagnosis. Most common symptoms are nonspecific such as fever, myalgia, easy fatigability, weight loss, and arthralgia.^7^ With advancing vascular damage, symptoms such as headache, claudication, carotidynia, dizziness, diminution of vision, angina, etc. can occur.^8^

Our patient who is a 29‐year‐old female, gravida 4 para 3 with 2 live issues with no abortion, presented to the department of obstetrics and gynecology (OBGYN) at 40 weeks of gestation with the finding of oligohydramnios (AFI <3 cm) at ultrasonography (USG) and was taken up for emergency lower segment caesarean section (LSCS). During her evaluation, she was found to have an absent radial pulse bilaterally. There were no intranatal or postnatal complications; both mother and baby were in good health. The patient was asymptomatic till the second postnatal day when she developed an acute onset headache and was further evaluated by the Medicine department. The most common complications associated with TA are reported to be hypertension, retinopathy, aortic regurgitation, and the development of aneurysm.^9^ Other devastating complications include stroke, intestinal ischemia,^10^ and limb gangrene.

Clinical examination, laboratory tests, artery biopsy, and imaging are the available modalities of diagnosis, imaging being the most important diagnostic method as clinical examination and laboratory tests are unreliable^11^ and biopsy of the large vessels is impractical.^6^ Absent pulses, blood pressure discrepancies between limbs, and arterial bruits are signs frequently present on examination. Our patient had absent bilateral radial, bilateral brachial, and left dorsalis pedis pulses. Blood pressure was not recordable in both upper limbs whereas it was measured as 160/100 mmHg, postdelivery, in bilateral lower limbs, suggestive of hypertension. A loud second pulmonary sound (P2) and carotid bruit were heard on auscultation.

Laboratory tests such as erythrocyte sedimentation rate (ESR) and C‐reactive protein (CRP) are not specific for TA and the elevation in ESR and CRP levels, seen in 80% and 60% of cases, respectively, only indicate underlying inflammation,^12^ although there are other markers such as interleukin‐6, RANTES^13^, ^14^ and tissue inhibitor of metalloproteinases‐1 (TIMP‐1),^15^ which show some specificity for TA. Based on the clinical manifestations of the patient and levels of acute phase reactants such as ESR and CRP, the Indian Takayasu Arteritis Clinical Activity Score was developed in 2010 (ITAS / ITAS‐A 2010).^16^ The blood workup of our patient showed an erythrocyte sedimentation rate of 130 mm at the end of an hour, which is elevated.

Conventional angiography is still the gold standard for diagnosis and treatment, although noninvasive techniques such as USG, computed tomography (CT), and magnetic resonance imaging (MRI) are routinely used.^12^ In TA, mural thickening, vascular stenosis or occlusion or dilatation, and surrounding edema can be made out.^17^ The Macaroni sign has been described as a pathognomonic sign for TA, which can be seen on the transverse cut of B mode USG.^18^ This sign represents a smooth, homogenous, and circumferential thickening of arterial wall, which is moderately echogenic. According to the new angiographic classification of Takayasu Arteritis, Takayasu conference 1994, TA is classified into 5 patterns. Type I affects aortic arch branches, type IIa affects the ascending aorta, the aortic arch, and its branches, type IIb affects the ascending aorta, the aortic arch, and its branches, as well as the thoracic descending aorta, type III affects the descending thoracic aorta, the abdominal aorta, and/or the renal arteries, type IV affects the abdominal aorta and/or renal arteries and type V is a combination of IIb and IV.^19^ Among the above‐mentioned patterns, type V is the most commonly seen.^20^, ^21^ For our patient, USG doppler showed diffuse intimal thickening of the proximal segment of the left common carotid artery with stenosis of 60% and CT aortogram was characteristic of type V TA (Numano classification), pointing towards the involvement of the entire aorta and branches of the aortic arch. Thereby, she was diagnosed to have active TA (ITAS‐A, score: 5, 2010).

The majority of the population affected by diseases of autoimmune etiology tend to be women and TA is one of the primary systemic vasculitides likely to occur among women in the childbearing age group.^22^ So, the interaction between TA and pregnancy becomes an area of attention. During pregnancy, the maternal immune system bends to accommodate the growing fetus, which is potentially a foreign tissue. The severity of autoimmune diseases or the risk of its relapse is reduced during pregnancy, especially in the case of those autoimmune diseases, which are mediated by Th1 cells, as it is postulated that there is a shift to Th2‐based immunity in pregnancy.^23^ This mechanism has not been well studied in the context of coexisting TA.

Studies have conflicting conclusions as to the effect of pregnancy on TA. It has been reported in patients with TA on assessing the level of inflammation and hemodynamic changes that ameliorate during pregnancy, with hypertension being one of the complications, which show deterioration, especially in the perinatal period.^24^, ^25^ The patient presented to us at term and was asymptomatic throughout pregnancy. Before the delivery, the pulse was palpable only in the right popliteal and dorsalis pedis. The first symptom of TA was headache, which manifested 2 days following delivery, although she had telltale signs of TA such as absent pulses and carotid bruit on examination even before delivery.

Coming to the effect of TA on pregnancy, various studies report different outcomes of pregnancy in patients with TA, but the majority of them worldwide and in India found that obstetric complications are highly likely and are higher in pregnancies after the diagnosis of TA was made than in those before its diagnosis.^2^ Vascular damage of TA combined with the normal physiological changes in hemodynamics during pregnancy can act as a catalyst for adverse maternal and fetal outcomes.^5^ The most common complication is hypertension in the form of gestational hypertension, preeclampsia, or eclampsia, though different studies show varying percentages. Vessel involvement is likely to reflect on the dynamics between TA and pregnancy as fetal complications such as IUGR and oligohydramnios are associated with renal artery stenosis, due to increased renin activity and resultant elevation of blood pressure and uteroplacental insufficiency.^26^ A fetal Doppler scan done in the 3rd trimester also showed abnormalities in the umbilical artery and middle cerebral artery circulation. The patient is currently on maintenance dose steroid therapy using prednisolone and Azathioprine, and is advised for regular follow‐up. The patient currently completed her 6‐month follow‐up. However, the relapse in chronic immune conditions like TA is common, and patients on immunosuppressive therapy usually have a good prognosis.

## 4 CONCLUSION

To conclude, our case brings out a term pregnancy with an uneventful antepartum period in a woman with extensive arterial damage by TA, though she was not aware of her condition. Peripartum flare‐up of the disease could have led to uteroplacental insufficiency as suggested by the third trimester Doppler scan, which led to acute oligohydramnios leading to a caesarean delivery, besides which there were no intrapartum complications.

## AUTHOR CONTRIBUTIONS

**Lokesh Koumar Sivanandam:** Conceptualization; data curation; methodology; supervision; writing – original draft. **Naresh Kumar Reddy:** Conceptualization; supervision; validation; writing – original draft; writing – review and editing. **Ratnasree Vaddepalli:** Conceptualization; data curation; methodology; validation; writing – original draft; writing – review and editing. **Vivek Sanker:** Conceptualization; data curation; methodology; validation; writing – original draft; writing – review and editing. **Anjuman Amina Mansoor:** Conceptualization; formal analysis; validation; writing – original draft; writing – review and editing. **Umang Gupta:** Conceptualization; data curation; methodology; validation; writing – original draft; writing – review and editing.

## FUNDING INFORMATION

None.

## CONFLICT OF INTEREST STATEMENT

None declared.

## ETHICS STATEMENT

Ethical approval was not required for the case report as per the country's guidelines.

## INFORMED CONSENT

Written informed consent was obtained from the patient to publish this report.

## Data Availability

The data that support the findings of this article are available from the corresponding author on reasonable request.
